# Relationship between cardiac microvascular dysfunction measured with 82Rubidium-PET and albuminuria in patients with diabetes mellitus

**DOI:** 10.1186/s12933-017-0652-1

**Published:** 2018-01-11

**Authors:** Louis Potier, Renata Chequer, Ronan Roussel, Kamel Mohammedi, Souad Sismail, Agnès Hartemann, Chloé Amouyal, Michel Marre, Dominique Le Guludec, Fabien Hyafil

**Affiliations:** 1 Department of Diabetology, Endocrinology and Nutrition, DHU-FIRE, HUPNVS, AP-HP, Paris, France; 2 Paris Diderot-Sorbonne Paris Cité University, Paris, France; 3 Centre de Recherche des Cordeliers, INSERM, U-1138, Paris, France; 4 Department of Nuclear Medicine, DHU-FIRE, HUPNVS, AP-HP, Paris, France; 5 Department of Diabetology-Metabolism, Pitié-Salpêtrière-Charles Foix Hospital, AP-HP, Paris, France; 6 Pierre and Marie Curie University (UPMC), Sorbonne University, Paris, France; 7 INSERM U-1166, Institute of Cardiometabolism and Nutrition (ICAN), Paris, France; 8 INSERM, U-1148, Paris, France

**Keywords:** Diabetic nephropathy, Coronary microvascular dysfunction, Albuminuria, Diabetes

## Abstract

**Background:**

Albuminuria is of one the strongest predictors of cardiovascular disease (CVD) in diabetes. Diabetes is associated with cardiac microvascular dysfunction (CMD), a powerful, independent prognostic factor for cardiac mortality. The aim of this study was to evaluate the relationship between CMD and microvascular complications in patients without known CVD.

**Methods:**

In this monocentric study, myocardial flow reserve (MFR) was measured with cardiac ^82^Rubidium positron emission tomography (Rb-PET) in 311 patients referred to nuclear medicine department of Bichat University Hospital for screening of coronary artery disease from 2012 to 2014. Patients with hemodynamically relevant stenosis on coronary angiography or myocardial ischemia on Rb-PET were excluded. Among patients with diabetes, MFR values were compared according to the presence of retinopathy and albuminuria.

**Results:**

Overall, 175 patients (118 with type 2 diabetes) were included. MFR was significantly lower in patients with diabetes compared with those without diabetes (2.6 ± 1.1 vs. 3.3 ± 1.7; p < 0.005). In patients with diabetes, MFR decreased progressively in relation to albumin urinary excretion (normoalbuminuria: 2.9 ± 1.1, microalbuminuria: 2.3 ± 1.0, macroalbuminuria: 1.8 ± 0.7; p < 0.0001). MFR was not significantly different in patients with vs. without retinopathy (2.4 ± 1.0 vs. 2.7 ± 1.1, p = 0.07). Microalbuminuria and macroalbuminuria remained strongly associated with impaired MFR after multiple adjustments [odds ratio 2.6 (95% CI 1.1–8.4) and 5.3 (95% CI 1.2–44.7), respectively]. This association was confirmed when analyses were restricted to patients with low levels of coronary calcifications on computed tomography.

**Conclusions:**

Impaired MFR was more frequent in patients with diabetes and was strongly associated with the degree of albuminuria suggesting that CMD and albuminuria might share common mechanisms.

**Electronic supplementary material:**

The online version of this article (10.1186/s12933-017-0652-1) contains supplementary material, which is available to authorized users.

## Background

Despite improvement in medical care, cardiovascular disease (CVD) remains the leading cause of death among patients with diabetes [1]. The onset of diabetic nephropathy, especially albuminuria, is a turning point in patients with diabetes. Indeed, albuminuria has been described as one of the strongest risk factor of coronary artery disease (CAD) or heart failure [2–6]. Similarly, diabetic retinopathy is closely related to cardiovascular mortality in epidemiological studies [7, 8]. However, the origin of the association between increased cardiovascular risk and diabetic microvascular complications remains unclear.

Cardiac microvascular dysfunction (CMD) is the impaired ability of microcirculation of the heart to adapt blood flow to meet oxygen demand. There is strong evidence that cardiac microvascular function of patients with either type 1 or type 2 diabetes is markedly impaired compared with the microvascular function of patients without diabetes [9, 10]. Moreover, CMD is an independent predictor of cardiac mortality and is associated with the development of cardiac dysfunction in patients with diabetes [11, 12]. Microvascular dysfunction appears early in diabetes course, and can be detected even in prediabetes [13].

Cardiac and renal microvascular impairment could share similar mechanisms. Previous studies suggested a relationship between albuminuria and CMD in patients with diabetes but were limited to comparison between normo vs. microalbuminuria in a small number of subjects [14, 15]. The aim of the present study was: (1) to confirm the higher prevalence of cardiac microvascular impairment in patients with diabetes and without overt CAD compared with those without diabetes, (2) to compare the severity of cardiac microvascular dysfunction in patients with diabetes according to albuminuria level.

## Methods

### Study population

We included 311 consecutive patients referred for the screening of CAD from October 2012 to December 2014 in the department of Nuclear Medicine of Bichat University Hospital, (Paris, France). Patients were eligible for inclusion if they had all the following criteria (1) age > 18; (2) body mass index (BMI) > 25 kg/m^2^ for men; (3) in patients with chest pain, estimated prevalence of CAD > 30% with the Diamond and Forrester clinical score [16] or, in absence of chest pain, presence of ≥ 3 cardiovascular risk factors [16]. Participants were not eligible if one of the following characteristics was present: (1) history of myocardial infarction, heart failure, or coronary revascularization; (2) coronary computed tomography angiography or coronary angiography within the prior 2 years; (3) life expectancy < 2 years; (4) serum creatinine > 200 µmol/L; (5) pregnancy or lactating.

All participants were imaged with ^82^Rubidium-positron emission tomography (Rb-PET) as part of the RUBIS study (clinicaltrials.gov: NCT01679886). Examinations were analyzed by experienced readers and classified as abnormal or normal for the presence of myocardial ischemia based on the results of the stress test and myocardial perfusion imaging (MPI). If MPI was classified as abnormal, an invasive coronary angiography was proposed to patients to assess coronary stenosis. Patients with hemodynamically relevant stenosis on coronary angiography (coronary stenosis ≥ 50% with fractional flow reserve ≤ 0.8, critical coronary stenosis without FFR and coronary occlusion), or patients with myocardial ischemia but who did not accept to undergo coronary angiography were excluded from this cohort to avoid the confounding effect of stenosis of epicardial coronary arteries. Patients with type 1 diabetes (n = 9) were excluded. National ethics committee approved the study protocol; all patients provided signed informed consent; the full description of the study design has been registered on clinicaltrials.gov (NCT01679886). In addition, the local ethical committee (CEERB Paris Nord-IRB 00006477—of HUPNVS, Paris 7 University, AP-HP) approved the retrospective access to clinical and biological data acquired in these patients.

### Clinical measurements

All clinical parameters were collected at the time of the Rb-PET. Blood pressure (BP) was measured with an automatic device (Dynamap^®^, France) using a cuff sized to the patient morphology, in a sitting position. Blood pressure was assessed three times consecutively at 3-min intervals, and the mean value was recorded. Diabetes was defined according to the WHO criteria. Retinopathy was assessed from medical record of patients and classified as absence of retinopathy, non-proliferative retinopathy or proliferative retinopathy. Blood samples were taken during the fasting state for routine biologic analyses [lipids, serum creatinine, glycated hemoglobin (HbA1c)] at the time of the Rb-PET during routine care. HbA1c was measured by HPLC and plasma creatinine by Jaffé method. Estimated glomerular filtration rate (eGFR) was calculated using the CKD-EPI equation [17]. Albumin was measured by nephelometry in 3 consecutive urine samples collected during outpatient visits in the 6 months before Rb-PET. Albumin to creatinine ratio (ACR) was classified as persistently normoalbuminuria, microalbuminuria, or macroalbuminuria, if their ACR ranged ≥ 2 times within the following values: < 3, 3–30, or > 30 mg/mmol, respectively.

### ^82^Rubidium-PET imaging protocol

#### PET imaging protocol

Patients were instructed to abstain from any products containing caffeine or methylxanthine-containing for the 24 h before pharmacological test and to discontinue beta-blocker drugs and calcium antagonists 48 h before pharmacological test. Pharmacological stress consisted of a standard infusion of dipyridamole during 4 min (0.7 mg/kg; maximal injected dose of 70 mg). For each PET acquisition, patients were injected intravenously with 82-Rubidium from a generator (CardioGen-82^®^; Princeton, NJ, USA) at a dose of 10 MBq/kg (minimal dose = 740 MBq; maximal dose = 1480 MBq). All patients were studied using a whole-body PET-CT scanner (Discovery ST Lightspeed 64, GE Healthcare, Milwaukee, Wisconsin). After a scout CT acquisition (120 kVp, 10 mA) used for proper patient positioning, a CT transmission scan with dose modulation (140 kVp, 20–30 mA, pitch 1.35) was acquired. PET acquisitions were performed in 3D mode and acquired in list-mode. Rest acquisition started immediately after initiation of Rb injection and lasted 8 min. After the rest acquisition, dipyridamole was injected over 4 min. Rb was administered 7 min after the start of dipyridamole injection. After completion of stress PET images, a post-emission CT transmission scan was repeated and used for attenuation correction of stress images.

#### Image reconstruction

Dynamic PET acquisitions were reconstructed using filtered back projection into 15 time frames (9 × 10 s, 3 × 30 s, 1 × 60 s, and 2 × 120 s; total, 8 min). Static and gated PET images were reconstructed with acquisitions between 2 and 8 min pi using an ordered subset expectation maximization algorithm (4 iterations, 24 subsets).

#### Image analysis

Stress and rest LVEF were measured using the 4DM-SPECT software (4DM; INVIA, LLC). Stress and rest myocardial blood flow was quantified on dynamic PET acquisitions using a one-compartment model for ^82^Rb uptake in the myocardium of the FlowQuant software [18]. In addition, rest myocardial blood flow was corrected for cardiac workload as described [19]. Myocardial flow reserve (MFR) was calculated as the ratio between stress myocardial blood flow (MBF) and corrected rest MBF. For clinical convenience, MFR was primarily displayed as a dichotomous variable using a cut-off point of 2.0 for an impaired ratio. MFR < 2.0 has been shown to be associated with worse cardiovascular outcomes in a general referral population [20, 21].

### Coronary calcium scoring

The extent of coronary artery calcification (CAC) was graded visually on the low-dose CT of the thorax acquired for attenuation correction of PET acquisitions using a 6-point scale by an operator (RC) blinded to the clinical measurements and PET results. This classification has been demonstrated to be reproducible and reflect CAC scores measured using dedicated gated cardiac CT acquisitions [22].

### Statistical analysis

Data are presented as mean (SD) values for quantitative parameters with normal distributions, or as median (quartile ranges) values for those with skewed distribution, or as numbers (percentages), for the qualitative parameters. Analyses of covariance (ANCOVA) were performed to compare quantitative variables, after adjustment for confounders (age, sex, eGFR, BMI, heart rate, systolic BP and smoking). Data were log-transformed before analyses, or non-parametric tests were used when the normality of the distribution was rejected by the Shapiro–Wilk W-test (ACR, triglycerides). Qualitative traits were analyzed by χ^2^, or Mantel–Haenszel tests. Multiple logistic regression analyses were used to evaluate association of MFR < 2.0 with ACR categories in four multi-adjusted models: model 1 adjusted on sex and age; model 2 adjusted for model 1 plus BMI, SBP, smoking status and LDL-cholesterol; model 3 adjusted for model 2 plus eGFR, HbA1c, duration of diabetes and model 4 adjusted for model 3 plus renin–angiotensin–aldosterone system (RAAS) blockers, beta blockers, and statins use. We compared MFR in patients with diabetes in different subgroups according age, sex, smoking status, diabetes duration, HbA1c, eGFR, BMI, presence of hypertension, antiplatelet agents and statins use. As sensitivity analyses, we used a threshold of 2.5 for impaired MFR and we excluded patients with CAC > 400 of our analyses. Authors had full access to all the data and take responsibility for its integrity and the data analysis. All statistical computations were performed using the JMP software (version 9, SAS Institute Inc., Cary, NC).

## Results

### Patient characteristics

**Fig. 1:**
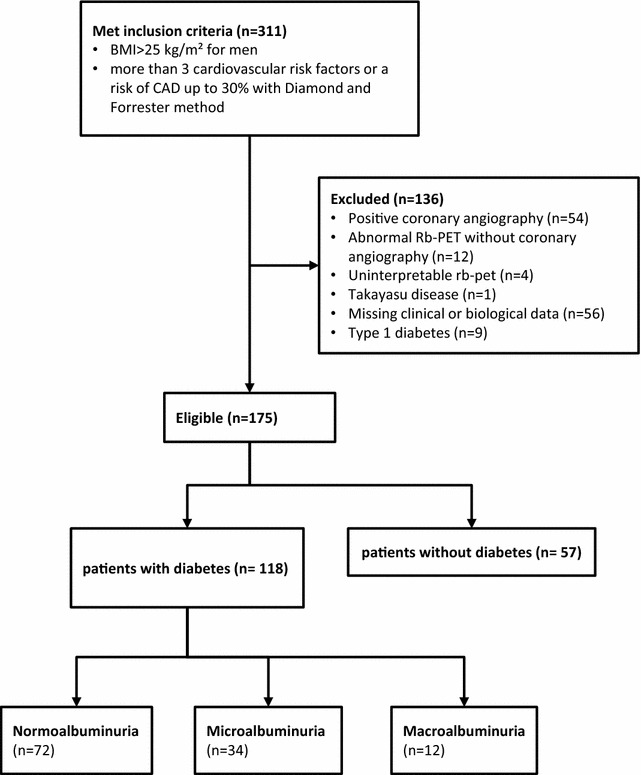
CONSORT flow chart showing the selection of patients included in this study

A total of 311 patients were included in the RUBIS study. Among them, 136 were excluded of these analyses as described in the flow chart (Fig. 1). Overall, 175 patients were included: 118 patients (69.0%) had type 2 diabetes (Fig. 1). Compared to non-diabetic individuals, patients with diabetes had a lower prevalence of family history of CVD (14.4% vs. 45.6%, p < 0.001), but a higher number of CV risk factors (including diabetes) (3.2 ± 0.6 vs. 2.8 ± 0.9, p < 0.05). They had a lower level of total cholesterol (4.3 ± 1.0 vs. 5.0 ± 0.1 mmol/L, p < 0.005) and LDL-cholesterol (2.4 ± 1.2 vs. 3.1 ± 0.9 mmol/L, p < 0.05).

Characteristics of participants with diabetes are shown in Table 1. Among them, 72 patients (61.10%) had normoalbuminuria, 34 patients (28.8%) microalbuminuria and 12 patients (10.2%) macroalbuminuria. No significant difference in rest and stress left ventricular ejection fraction (LVEF) was detected between patients without and with diabetes and between normo, micro and macroalbuminuric patients (Table 2). A smaller increase in LVEF during pharmacological stress was measured between patients with diabetes compared with patients without diabetes (5.6 ± 5.9% vs. 8.1 ± 7.8%, respectively; p < 0.05).

**Table 1:** Clinical characteristics of patients with diabetes

	All	Normo albuminuria	Micro albuminuria	Macro albuminuria	p
n (%)	118	72 (61.1)	34 (28.8)	12 (10.2)	
Age (years)	59.6 ± 8.8	59.5 ± 9.4	60.5 ± 8.4	57.7 ± 5.6	0.62
Female	62 (52.54)	38 (52.3)	19 (55.9)	5 (41.7)	0.73
BMI (kg/m^2^)	32.8 ± 7.0	32.8 ± 7.0	33.3 ± 7.6	31.2 ± 4.8	0.71
Systolic BP (mmHg)	131.2 ± 1.5	128.0 ± 14.5	136.4 ± 15.5	136.2 ± 13.6	0.02
Diastolic BP (mmHg)	75.1 ± 1.4	74.7 ± 11.2	76.0 ± 21.2	75.4 ± 8.0	0.94
Heart rate (bpm)	79.2 ± 1.3	77.3 ± 12.3	82.4 ± 12.0	82.4 ± 12.3	0.23
Diabetes duration (years)	12.8 ± 9.3	11.3 ± 8.7	13.3 ± 9.3	19.9 ± 9.5*	0.018
Retinopathy	34 (31.5)	13 (20.0)	13 (41.9)	8 (66.7)*	0.002
Former or current smoking	25 (21.2)	18 (25)	6 (17.7)	1 (8.3)	0.33
Hypertension	105 (88.98)	62 (86.1)	31 (91.2)	12 (100)	0.22
Family history of cardiovascular disease	17 (14.4)	9 (12.5)	7 (20.6)	1 (8.3)	0.54
Number of cardiovascular risk factor	3.2 ± 0.6	3.1 ± 0.6	3.2 ± 0.7	3.2 ± 0.4	0.92
HbA1c (%)	8.4 ± 1.8	8.2 ± 1.7	8.4 ± 1.6	9.9 ± 1.8*	0.01
Total cholesterol (mmol/L)	4.3 ± 1.0	4.3 ± 1.0	4.3 ± 1.1	4.5 ± 1.2	0.73
Triglycerides (mmol/L)	1.9 (1.6–2.2)	17 (1.4–2.2)	1.9 (1.3–2.5)	3.0 (2.0–3.9)*	0.004
HDL-cholesterol (mmol/L)	1.2 ± 0.37	1.2 ± 0.4	1.1 ± 0.4	1.0 ± 0.2	0.23
LDL-cholesterol (mmol/L)	2.4 ± 1.0	2.4 ± 1.3	2.3 ± 1.0	2.4 ± 0.9	0.89
eGFR (mL/min/1.73 m)	81.1 ± 24.4	83.8 ± 20.8	83.5 ± 24.7	58.6 ± 32.5*	0.003
ACR (mg/mmol)	17.8 (7.3–28.3)	1.2 (1.1–1.43)	10.0 (7.1–12.9)	139.0 (57.0–221.0)*	< 0.001
RAAS blockers	81 (73.6)	48 (69.6)	22 (73.3)	12 (100)*	0.03
Beta-blockers	27 (25)	14 (20.6)	7 (24.1)	6 (54.6)	0.08
Anti platelet agents	45 (42.1)	26 (38.8)	10 (35.7)	9 (75)	0.04
Statins	88 (80)	50 (74.6)	26 (83.9)	12 (100)*	0.03
Metformine	96 (85.7)	59 (86.8)	29 (90.6)	8 (66.7)	0.2
Sulfonylureas	45 (42.5)	29 (44.6)	14 (46.7)	2 (18.2)	0.2
DPP4 inhibitors	28 (26.7)	14 (21.5)	11 (37.9)	3 (27.3)	0.3
GLP1 analogs	14 (13.5)	8 (12.5)	5 (17.2)	1 (9.1)	0.7
Insulin	66 (60)	36 (53.7)	18 (58.1)	12 (100)*	0.01

Data are n (%), mean ± SD or geometric mean (IQR)

*BMI* body mass index, *eGFR* estimated glomerular filtration rate, *ACR* albumin creatinin ratio, *RAAS* renin angiotensin aldosterone system

* p < 0.05 vs. normoalbuminuria

**Table 2:** Cardiac Rubidium-PET measurements in patients with diabetes

Variable	All	Normo albuminuria	Micro albuminuria	Macro albuminuria	ANOVAp
MFR	2.6 ± 1.1	2.9 ± 1.1	2.3 ± 1.0*	1.8 ± 0.7*	< 0.001
MFR < 2.0	37 (31.4)	14 (19.0)	154(41.2)*	9 (75.0)*^†^	< 0.001
MFR < 2.5	56 (47.5)	29 (40.3)	18 (52.9)	9 (75.0)*^†^	0.04
CAC > 400	9 (7.9)	5 (7.1)	3 (8.8)	1 (10)	0.85
Rest LVEF	56.1 ± 9.2	56.4 ± 9.0	56.2 ± 10.6	54.1 ± 6.0	0.81
Stress LVEF	61.6 ± 8.9	62.2 ± 8.5	60.1 ± 10.4	59.6 ± 6.8	0.67
Difference in LVEF between stress and rest	5.6 ± 5.9	6.1 ± 5.9	4.6 ± 6.2	5.5 ± 5.0	0.43

Data are n (% of the total number of patients of each group). mean ± SD

*LVEF* left ventricular ejection fraction, *MFR* myocardial flow reserve, *CAC* coronary arteries calcium

* p < 0.005 vs. normoalbuminuria, ^†^ p < 0.005 vs. microalbuminuria

### Myocardial flow reserve measurements

MFR was significantly lower in patients with diabetes compared with patients without diabetes (2.6 ± 1.1 vs. 3.3 ± 1.7, respectively; p < 0.005). The prevalence of impaired MFR was higher in patients with diabetes compared with patients without diabetes (31.4% vs. 15.8% respectively, p < 0.05). The difference in MFR between patients with and without diabetes remained significant after adjustment for age, sex, eGFR, BMI, heart rate, systolic blood pressure and smoking (2.5 ± 0.1 vs. 3.0 ± 0.2, respectively, p < 0.001). No difference in MFR was detected between normoalbuminuric patients with diabetes and patients without diabetes (2.9 ± 1.1 vs. 3.3 ± 1.7 respectively; p = 0.13).

**Fig. 2:**
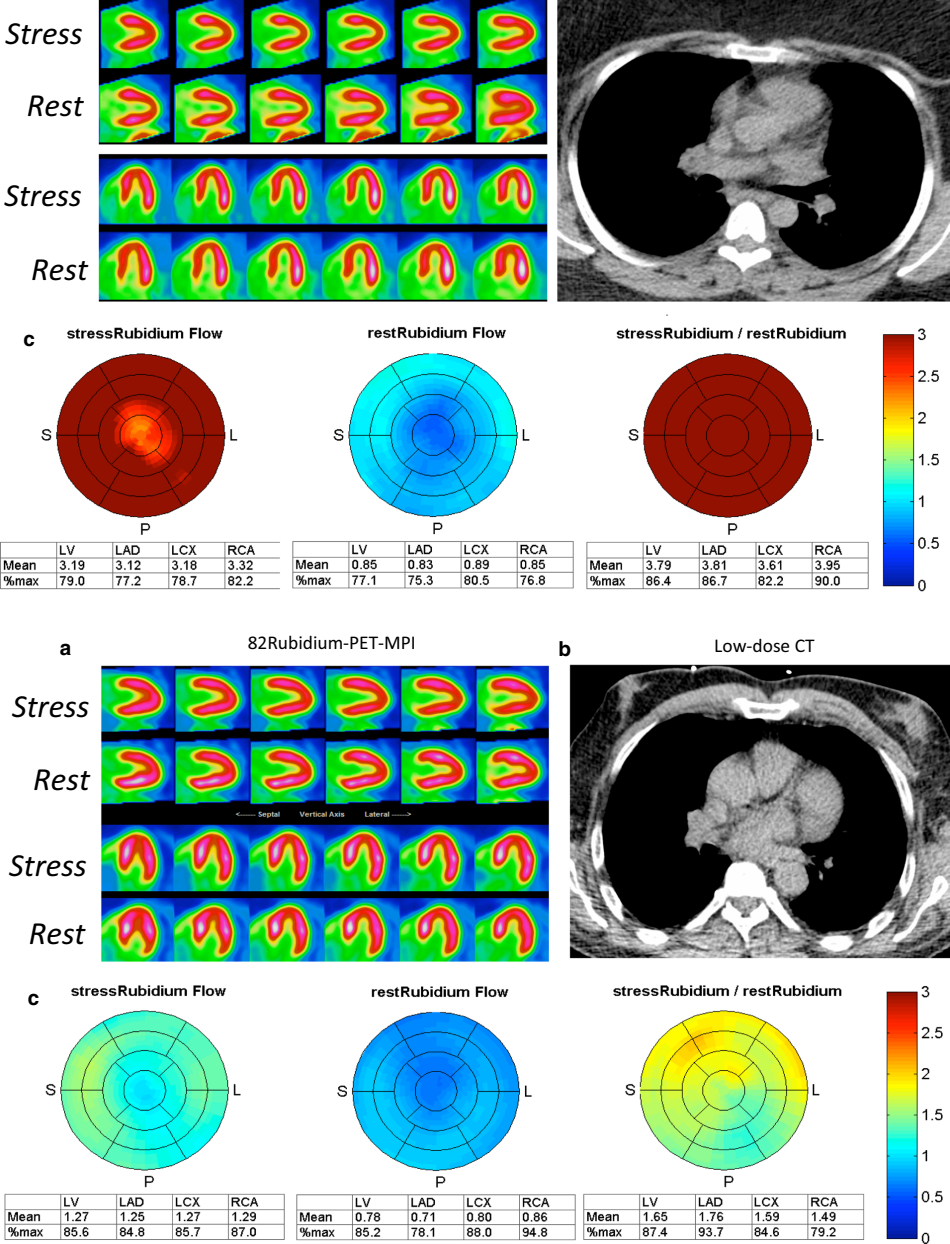
Representative examples of Rubidium-PET myocardial perfusion imaging (MPI) of diabetic patients with normoalbuminuria (**A**) and macroalbuminuria (**B**). No myocardial ischemia was present on Rb-PET MPI (a), nor coronary calcification on the low-dose CT used for attenuation correction of PET images (b) in both patients. Quantification of myocardial blood flow (MBF) with Rb-PET evidenced the presence of a global normal stress MBF and myocardial flow reserve in favor of a normal cardiac microvascular function (MFR = 5.1) in normoalbuminuric patient and a global low stress MBF and myocardial flow reserve in favor of cardiac microvascular dysfunction (MFR = 1.6) in macroalbuminuric patient (c)

Among patients with diabetes, there was a significant decrease in MFR with increasing ACR (Table 2). MFR in microalbuminuric and macroalbuminuric patients was significantly lower compared with normoalbuminuric patients (normoalbuminuric 2.9 ± 1.1, microalbuminuric 2.3 ± 1.0, macroalbuminuric 1.8 ± 0.7; p < 0.05 vs. normoalbuminuric patients for both) (Fig. 2). Relationship between ACR as a continuous variable and MFR was significant (r^2^ = 0.11, p < 0.001). The prevalence of impaired MFR increased with the degree of albuminuria (19.0, 41.2 and 75.0% in normo-, micro-, macroalbuminuric patients respectively, p < 0.001). Similar results were observed with a threshold of 2.5 for impaired MFR (40.3, 52.9 and 75.0% in normo-, micro-, macroalbuminuric patients respectively; p < 0.05). We also observed a trend toward a lower MFR in patients with retinopathy compared with patients without retinopathy (2.4 ± 1.0 vs. 2.7 ± 1.1 respectively, p = 0.09) and MFR tended to decrease with the severity of retinopathy (non-proliferative retinopathy: 2.6 ± 1.1, proliferative retinopathy: 2.1 ± 1.0; p = 0.18). No association was found between HbA1c or diabetes duration and MFR (p = 0.18 and p = 0.50, respectively).

**Fig. 3:**
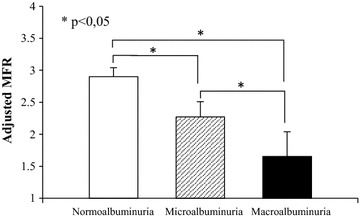
Adjusted MFR according degree of albuminuria (estimated marginal means with standard of error means). Adjusted for age, sex, BMI, eGFR, duration of diabetes, HbA1c, and systolic BP. *p < 0.05

After adjustment for systolic BP, duration of diabetes, eGFR, HbA1c, triglycerides, RAAS blockers, antiplatelet agents, statins and insulin use, the differences in MFR between groups of albuminuria remained significant (Fig. 3). In multiple logistic regression analyses using different adjusted model, microalbuminuria and macroalbuminuria were associated with a three and eightfold increase of impaired MFR respectively (Table 3). No association was observed between MFR and eGFR (r^2^ = 0.004, p = 0.82). However, in subgroups of patients with mild to moderate chronic kidney disease (stage 2 and 3: eGFR 60–89 and 30–59 mL/min, respectively), MFR was significantly lower in patients with micro and macroalbuminuria compared with normoalbuminuric patients (stage 2: normoalbumuria = 2.9 ± 1.3, microalbuminuria = 2.2 ± 1.0, macroalbuminuria = 1.9 ± 0.7, p < 0.05. Stage 3: normoalbumuria = 3.2 ± 0.9, microalbuminuria = 2.9 ± 1.0, macroalbuminuria = 1.6 ± 1.0, p < 0.05).

**Table 3:** Risk of impaired MFR with microalbuminuria and macroalbuminuria according to multiple adjusted logistic regression model vs. normoalbuminuria as the reference group

	Microalbuminuria			Macroalbuminuria		
	OR	95% CI	p	OR	95% CI	p
Model 1	2.9	1.2–7.1	0.02	13.3	3.4–67.0	0.001
Model 2	2.6	1.1–7.2	< 0.05	4.9	1.0–29.5	0.06
Model 3	2.6	1.1–8.4	< 0.05	5.3	1.2–44.7	0.03
Model 4	2.2	0.9–8.5	0.06	9.8	1.4–313.2	< 0.05

Model 1: adjusted on sex and age. Model 2: adjusted on model 1 + BMI, SBP, smoking status and LDL-cholesterol. Model 3: model 2 + eGFR, HbA1c, and duration of diabetes. Model 4: model 3 + RAAS blockers, beta blockers, and statins use

We compared MFR in patients with diabetes in different subgroups (Additional file 1: Table S1). Despite a few numbers of subjects without hypertension (n = 13), no significant difference in MFR was observed between patients with and without hypertension (2.6 ± 1.1 vs. 2.6 ± 0.9 respectively, p = 0.80). However, considering all patients, we observed a progressive decrease in MFR in association with the presence of hypertension, diabetes or both (no diabetes, no hypertension: 4.2 ± 2.8; no diabetes, hypertension: 3.0 ± 1.1; diabetes, no hypertension: 2.6 ± 1.1; diabetes and hypertension: 2.6 ± 0.9, p < 0.001).

### CAC measurements

Among patients with diabetes, only nine patients (7.9%) had a CAC estimated > 400. There was no association between severity of albuminuria and prevalence of a CAC > 400 (Table 2). Rate of CAC > 400 was similar in patients with MFR ≤ 2.0 and MFR > 2.0 (3.5% vs. 4.4% respectively, p = 0.33). MFR were similar in patients with high (> 400) or and low (≤ 400) CAC score (2.4 ± 1.0 and 2.7 ± 1.1 respectively, p = 0.41). After exclusion of patients with CAC > 400, association between MFR and degree of albuminuria remained significant (p < 0.005).

## Discussion

In this study, we found a significant association between the severity of cardiac and renal microvascular disease in patients with diabetes without overt cardiovascular disease. We found a higher prevalence of impaired intra-myocardial microcirculatory function in diabetic patients with normal LV function and without myocardial ischemia compared with non-diabetic patients. In addition, we observed a clear reduction of MFR in patients with the highest level of albuminuria. This association was independent of other cardiovascular risk factors or potential confounding factors.

### Quantification of myocardial blood flow with Rb-PET

PET myocardial perfusion stress test (MPS) offers higher diagnostic performance for the detection of myocardial ischemia than SPECT-MPS, particularly in obese patients, thanks to a more accurate attenuation correction of the signal [23]. In addition to the evaluation of the presence and extent of myocardial ischemia, PET-MPS allows for the quantification of MBF during pharmacological stress and at rest. Quantification of MBF helps for the identification of balanced myocardial ischemia in patients with severe three-vessel CAD, but can also be reduced in patients with only moderate or absent CAD. MFR values can be extracted easily from the MPS-PET acquisitions and might represent an interesting additional marker of cardiovascular risk in patients with diabetes.

### Diabetes and cardiac microvascular dysfunction

Our findings show a higher prevalence of impaired MFR in patients with diabetes compared with those without diabetes. This is in line with previous studies showing that diabetes is associated with CMD [10]. The increased prevalence and severity of CMD in patients with diabetes may account for a major role for the worse cardiovascular risk observed among patients with diabetes compared with non-diabetic patients. Therefore, there is consistent evidence that impaired cardiac microvascular function is associated with adverse CV prognosis [21, 24]. Murthy et al. showed in a large study of 2783 patients that patients with diabetes and impaired MFR without CAD experienced a high risk of cardiac death comparable to that of non-diabetic patients with known CAD [11]. Similarly, other studies have shown an association between CMD and heart failure with preserved ejection fraction [12, 25]. Diabetes-induced CMD could explain, at least partially, the heavy burden of cardiac disease associated with diabetes.

The role of optimal glycemic control in the development of CMD remains largely discussed. Huang et al. found a twofold higher prevalence of CMD in patients with T2D with HbA1c > 7.1% compared with patients with good glycemic control, but this study was performed only in 24 patients [26]. In our study, we did not find any association between MFR and HbA1c. In our study, we did not find such an association between MFR and HbA1c. HbA1c value measured at the time of Rb-PET might, however, not reflect the long term quality of glycemic control over the duration of diabetes. Similarly, the prevalence of impaired MFR did not increase with the duration of diabetes in our study.

### Renal and cardiac microvascular injury in diabetes

In the current study, we showed that the levels of albuminuria were independently associated with the severity of CMD in patients with diabetes. Recently, Scholten et al. [15] showed similar results in 60 patients with type 2 diabetes. However, they only compared patients without vs. with albuminuria and found no difference of MFR between normoalbuminuric and albuminuric patients after adjustment for age, BMI, eGFR and heart rate, maybe due to the small size of sample. Here, in a larger cohort of patients with diabetes, we observed that the risk of impaired MFR increased significantly with the degree of albuminuria after multiple adjustments for confounding variables. Furthermore, micro- and macro-albuminuria were the main variables found to be significantly associated with the prevalence of impaired MFR. Unexpectedly, we found in this study no association between MFR and eGFR. Similarly, despite a trend toward a relationship between eGFR and MFR, Chade et al. [27] found that this relationship disappeared after adjustment for several risk factors. Moreover, in our study, MFR was lower in patients with albuminuria even in patients with mild to moderate chronic kidney disease (CKD). This is in line with the study from Imamura et al. [28] which showed that in patients with mild-to-moderate CKD but no diabetes, patients with albuminuria had impaired MFR compared with those without. It has been suggested for decades that albuminuria reflects widespread cardiovascular damage [29, 30]. Moreover, post hoc analysis of ADVANCE study in type 2 diabetic subjects has shown that albuminuria and eGFR were independent risk factors for cardiovascular events [30]. It is likely that albuminuria and reduced GFR may be markers of different pathologic processes. Taken together, these suggest that albuminuria, rather than alteration of renal filtration, was closely link to CMD. One could be hypothesized that CMD and albuminuria share common hyperglycemia-induced pathways including low-grade pro-inflammatory state, chronic ischemia, oxidative stress and activation of the renin-angiotensin-system.

If CMD shares common pathological pathways with diabetic nephropathy, CMD might benefit from the same treatments that have previously demonstrated beneficial effects at reducing glomerular lesions. In fact, angiotensin converting enzyme inhibitors and angiotensin receptor blockers have shown to be protective in diabetic nephropathy [31–33], but the effect of each of these treatments on CMD in patients with diabetes are conflicting. In contrast, spironolactone has been found to improve coronary microvascular function in patients with T2D [34]. Interestingly, dapagliflozin, a SGLT2 inhibitor, improved peripheral micro- and macro-vascular function [35], that might be an explanation for the reduction of both renal and cardiovascular events observed in diabetic patients treated with SGLT2 inhibitors [36, 37].

### Relationship between cardiac microvascular dysfunction and diabetic retinopathy

Although a trend was observed, we found no significant association between MFR and diabetic retinopathy, possibly in relation to the small size of our population. However, Akasaka et al. [38] showed that MFR was significantly reduced in patients with type 2 diabetes and diabetic retinopathy, especially in advanced retinopathy. A similar result was found in young patients with type 1 diabetes [39]. Recently, a positive association has been shown between MFR and cardiac autonomic function [40]. These results suggest that the association found here between diabetic nephropathy and CMD could be extended to retinopathy or maybe others diabetic microvascular complications, which all may share similar mechanisms.

### Strengths and limitations

Noninvasive measures of MFR integrate the hemodynamic effects of epicardial coronary stenosis and the presence of CMD. It is therefore important to exclude patients with extensive and diffuse CAD multi-variate analysis. After exclusion of patients with coronary stenosis or regional myocardial ischemia, 184 patients remained in the analysis providing more statistical power to identify associations than in the former study of Scholten performed in only 60 patients with type 2 diabetes patients [15]. Despite these precautions, we cannot exclude that the presence of a diffuse atherosclerotic process in coronary arteries might have played a role in the decrease of MFR observed in some of the patients. The association between CMD and albuminuria remained, however, significant after exclusion of patients with CAC > 400 suggesting a minor role in the relationship observed between CMD and albuminuria evidenced in this study. Second, the relatively small sample size increased the likelihood of a type II error. Nevertheless, the results remained significant after multivariable adjustment. Third, future studies need to evaluate more precisely and prospectively the time course of CMD in diabetic patients.

## Conclusions

We observed a strong association between the intensity of MFR reduction measured with Rb-PET and the severity of diabetic nephropathy in patients with diabetes. These results suggest that CMD and microvascular nephropathy might share common pathways. Further studies are needed to better understand the time course of CMD and its impact on cardiac function and the occurrence of cardiovascular events in diabetic patients.

## Additional file

**Additional file 1.** MFR according to different variables. eGFR: estimated glomerular filtration rate. BMI: body mass index.

## Abbreviations

ACR: albumin to creatinine ratio
BMI: body mass index
CAC: coronary artery calcium
CAD: coronary artery disease
CKD: chronic kidney disease
CMD: cardiac microvascular dysfunction
CVD: cardiovascular disease
eGFR: estimated glomerular filtration rate
FFR: fractional flow reserve
LVEF: left ventricular ejection fraction
MBF: myocardial blood flow
MPS: myocardial perfusion stress
MFR: myocardial flow reserve
RAAS: renin–angiotensin–aldosterone system
Rb-PET: ^82^Rubidium-positron emission tomography
