# Supplementary material for: Relationship between cardiac microvascular dysfunction measured with 82Rubidium-PET and albuminuria in patients with diabetes mellitus
Source: Cardiovasc Diabetol. 2018 Jan 11;17:11. doi: 10.1186/s12933-017-0652-1 (PMC5763541; doi:10.1186/s12933-017-0652-1)
Supplement: Supplementary file 1 — Additional file 1. MFR according to different variables. eGFR: estimated glomerular filtration rate. BMI: body mass index. [file 12933_2017_652_MOESM1_ESM.docx]

**Additional file**

| **Variables** |  | **MFR** | **p** |
| --- | --- | --- | --- |
| **Age (years)** | ≤60 | 2.7±1.1 | 0.46 |
|  | >60 | 2.6±1.1 |  |
| **Sex** | Female | 2.5±0.9 | 0.08 |
|  | Male | 2.8±1.2 |  |
| **Smoking** | Yes | 2.6±1.1 | 0.94 |
|  | No | 2.6±1.1 |  |
| **Diabetes duration (years)** | ≤10 | 2.6±1.0 | 0.96 |
|  | >10 | 2.6±1.1 |  |
| **HbA1c (%)** | ≤7,5 | 2.8±1.3 | 0.17 |
|  | >7.5 | 2.5±0.9 |  |
| **eGFR (ml/min)** | >60 | 2.6±1.1 | 0.97 |
|  | ≤60 | 2.6±1.1 |  |
| **BMI (kg/m2)** | <30 | 2.8±1.3 | 0.27 |
|  | ≥30 | 2.5±1.0 |  |
| **Hypertension** | Yes | 2.6±1.1 | 0.23 |
|  | No | 3.0±1.3 |  |
| **Antiplatelet agents** | Yes | 2.6±1.3 | 0.87 |
|  | No | 2.6±0.9 |  |
| **Statins** | Yes | 2.6±1.2 | 0.85 |
|  | No | 2.6±0.8 |  |

**Table S1**: MFR according to different variables. eGFR: estimated glomerular filtration rate. BMI: body mass index.
